# Supplementary material for: Evolution of the climatic tolerance and postglacial range changes of the most primitive orchids (Apostasioideae) within Sundaland, Wallacea and Sahul
Source: PeerJ. 2016 Aug 31;4:e2384. doi: 10.7717/peerj.2384 (PMC5012329; doi:10.7717/peerj.2384)
Supplement: Table S1 [file peerj-04-2384-s001.doc]

S1 Table. List of localities used in ENM analysis.

| Species | Coordinates | | Collection | Herbarium |
| --- | --- | --- | --- | --- |
| *A. nuda* | -1,21667 | 110,1 | Laman & al. 250 | K |
| *A. nuda* | 4,096389 | 114,7119 | Boyce & al. 306 | K |
| *A. nuda* | 4,565278 | 115,165 | Poulsen 312 | K |
| *A. nuda* | 4,083333 | 114,9167 | Nielsen 698 | K |
| *A. nuda* | 5,33 | 111,05 | Vermeulen & Lamb 715 | K |
| *A. nuda* | 4,509444 | 115,6447 | Wood 728 | K |
| *A. nuda* | 5,33 | 117,05 | Vermeulen & Lamb 737 | K |
| *A. nuda* | 4,840833 | 115,7606 | Wood 794 | K |
| *A. nuda* | 4,840278 | 115,7606 | Wood 794 | K |
| *A. nuda* | 4,21 | 115,42 | Vermeulen & Duistermaat 935 | K |
| *A. nuda* | -4,34 | 115,15 | Simpson & Marsh 2252 | K |
| *A. nuda* | 1,716667 | 110,4664 | Purseglovo 5070 | K |
| *A. nuda* | 4,483333 | 115,1833 | Dransfield & al. 7208 | K |
| *A. nuda* | 6,012778 | 116,6353 | Beaman & al. 7424 | K |
| *A. nuda* | 4,9625 | 118,175 | Beaman 11638 | K |
| *A. nuda* | 3,95 | 114,7833 | Jermy 13686 | K |
| *A. nuda* | 5,034444 | 118,3364 | Perumal & Dewon 34994 | K |
| *A. nuda* | 1,590833 | 110,1703 | Lee Meng Hock 54175 | K |
| *A. nuda* | 1,995 | 112,9331 | Runi & al. 63767 | K |
| *A. nuda* | 5,866111 | 117,9683 | Sundaling 91079 | K |
| *A. nuda* | 5,498333 | 117,7014 | Sundaling 92365 | K |
| *A. nuda* | 5,795556 | 117,2728 | Sundaling & al. 102380 | K |
| *A. nuda* | 4,433333 | 115,75 | Lim & Antuk 143572 | K |
| *A. nuda* | 4,39 | 102,08 | Chua & al. FRI38850 | K |
| *A. nuda* | 4,681667 | 101,2994 | Robinson s.n. | K |
| *A. nuda* | 5,783333 | 100,4342 | Corner & Mauen s.n. | K |
| *A. nuda* | 5,783333 | 100,4342 | Robinson & Kloss s.n. | K |
| *A. odorata* | 6,116111 | 116,6581 | Andau 245 | K |
| *A. odorata* | 7,166667 | 116,7167 | Tadong 433 | K |
| *A. odorata* | 8,494167 | 99,73 | Kerr 0586 | K |
| *A. odorata* | 6,008611 | 116,5947 | Chew & al. 1351 | K |
| *A. odorata* | 6,066667 | 116,7 | Chew & al. 1670 | K |
| *A. odorata* | -12,14 | 120,46 | Vermeulen 2425 | K |
| *A. odorata* | 6,1625 | 116,5281 | Lugas 2436 | K |
| *A. odorata* | 6,0225 | 116,5397 | Chew & al. 2764 | K |
| *A. odorata* | 0,933333 | 100,4333 | Burley & al. 2938 | K |
| *A. odorata* | 6,236944 | 99,80611 | Robinson 6280 | K |
| *A. odorata* | 6,185833 | 116,5117 | Beaman & al. 9792 | K |
| *A. odorata* | 5,77 | 116,35 | Beaman & Christenson 10694 | K |
| *A. odorata* | 27,49833 | 104,84 | Henry 13738 | K |
| *A. odorata* | 16,1325 | 107,7378 | Averyanov & al. HAL10929 | MO |
| *A. odorata* | 17,18028 | 106,4894 | Averyanov & al. HAL11519 | MO |
| *A. odorata* | 17,69722 | 105,765 | Averyanov & al. HAL11624 | MO |
| *A. odorata* | 21,75667 | 105,8761 | Lôc & al. HAL9093 | HN, LE, MO |
| *A. odorata* | 19,05194 | 104,6158 | Averyanov & al. HLF6632 | MO |
| *A. odorata* | 19,07806 | 104,3475 | Averyanov & al. HLF6986 | MO |
| *A. odorata* | 6,350278 | 99,80056 | Corner s.n. | K |
| *A. odorata* | 12,18333 | 108,7167 | Averyanov VH4174 | MO database |
| *A. odorata* | 12,2 | 108,7333 | Averyanov VH4378 | MO database |
| *A. wallichii* | -17 | 145,7 | Lyons 32 | BRI |
| *A. wallichii* | 4,561944 | 115,1603 | Poulsen 122 | K, AAU |
| *A. wallichii* | -12,7 | 143,1 | Wrigley 261 | BRI |
| *A. wallichii* | 0,844444 | 131,2897 | Pleyte 340 | K |
| *A. wallichii* | -12,8 | 143,3 | Dockrill 454 | BRI |
| *A. wallichii* | -16,8 | 145,6 | Zich 634 | CANBR |
| *A. wallichii* | -0,6167 | 112,25 | Church 673 | CANBR |
| *A. wallichii* | -16,9 | 145,8 | Jago 793 | CNS |
| *A. wallichii* | -3,3075 | 114,6022 | Motley 840 | K |
| *A. wallichii* | 9,385278 | 98,55028 | Hoed & Kostermans 934 | K |
| *A. wallichii* | -16,9 | 145,7 | Dowe 1028 | CNS |
| *A. wallichii* | -7,61944 | 112,6167 | Comber 1054 | K |
| *A. wallichii* | -16,05 | 145,3 | Blaxell 1087 | K |
| *A. wallichii* | -4,03333 | 103,9667 | Vogel 1284 | K |
| *A. wallichii* | 9,366111 | 118,335 | Dransfield & al. 1293 | K |
| *A. wallichii* | 6,381944 | 80,50222 | Waas 1949 | K |
| *A. wallichii* | -16,3917 | 145,3 | Brass 2075 | BRI |
| *A. wallichii* | -17 | 145,8 | Costion 2114 | CNS |
| *A. wallichii* | 1,573333 | 103,5111 | SBGO 2118 | K, SING |
| *A. wallichii* | 8,785 | 117,7067 | Co 3398 | K, US |
| *A. wallichii* | 6,663056 | 100,3219 | Kerr 3698 | K |
| *A. wallichii* | -18,4 | 146,0833 | Jones & Clements 4094 | K |
| *A. wallichii* | 14,67694 | 108,3978 | Harder & Nguyên 4690 | MO database |
| *A. wallichii* | 11,98667 | 102,3081 | Larsen 4951 | K |
| *A. wallichii* | 13,3975 | 120,9958 | Coode 5415 | K |
| *A. wallichii* | 13,11611 | 121,0794 | Merrill 5639 | K |
| *A. wallichii* | -0,86139 | 134,0619 | Sands & al. 6138 | K |
| *A. wallichii* | 12,04778 | 102,3236 | Geesink & al. 6577 | K |
| *A. wallichii* | 21,97222 | 104,3378 | Harder & al. 6626 | MO database |
| *A. wallichii* | 22,04194 | 103,9756 | Harder 6905 | MO database |
| *A. wallichii* | 5,986389 | 116,6617 | Beaman & al. 7414 | K |
| *A. wallichii* | -5,58722 | 144,5531 | Womersley & Millar 7691 | K |
| *A. wallichii* | -16,1792 | 145,4239 | Gray 08453 | K |
| *A. wallichii* | -12,7 | 143,2 | Gray 8621 | BRI |
| *A. wallichii* | 6,133333 | 116,4833 | Beaman & al. 8709 | K |
| *A. wallichii* | -18,3 | 146,1839 | Everist 9685 | BRI |
| *A. wallichii* | -8,8775 | 147,7353 | Carr 10655 | K |
| *A. wallichii* | 8,053056 | 98,57194 | Hansen 12400 | K |
| *A. wallichii* | -18,4 | 146,4 | Cumming 12782 | BRI |
| *A. wallichii* | -16,5 | 145,5 | Carlquist 15537 | BRI |
| *A. wallichii* | -18,3931 | 146,3197 | Blake 18840 | K |
| *A. wallichii* | -13,9 | 143,3 | Jones 18887 | BRI |
| *A. wallichii* | -13,7 | 143,4 | Jones 18971 | BRI |
| *A. wallichii* | 2,428056 | 103,8111 | Banying 19171 | K |
| *A. wallichii* | 6,975833 | 116,7733 | Meijer 19893 | K |
| *A. wallichii* | -7,25 | 146,5 | Kairo 25569 | K |
| *A. wallichii* | -10,0628 | 151,1944 | Brass 25570 | K |
| *A. wallichii* | 12,86667 | 102,1667 | Larsen & al. 32056 | K |
| *A. wallichii* | 9,416667 | 98,41667 | Larsen & Larsen 33304 | K |
| *A. wallichii* | 9,385278 | 98,55028 | Larsen & al. 33304 | K |
| *A. wallichii* | 5,633333 | 117,2 | Leopold & Henry 58822 | K |
| *A. wallichii* | -7,25 | 146,5 | Katik & Larivita 62034 | K |
| *A. wallichii* | 5,984444 | 116,0836 | Pikkoh 67642 | K |
| *A. wallichii* | 5,495278 | 117,7731 | Madani 81708 | K |
| *A. wallichii* | -7,95389 | 147,5986 | Bau 82940 | K |
| *A. wallichii* | 5,436944 | 117,185 | Sundaling 90334 | K |
| *A. wallichii* | -1,48333 | 112,5167 | Argent & al. 93171 | K |
| *A. wallichii* | 5,626389 | 117,1308 | Rahim & al. 93282 | K |
| *A. wallichii* | 6,125278 | 116,6667 | Mansus & al. 122201 | K |
| *A. wallichii* | 5,616111 | 117,1161 | Majawat & al. 132958 | K |
| *A. wallichii* | -16 | 145,4 | Ford AF2584 | BRI |
| *A. wallichii* | -14,3 | 143,1 | Roberts DLJ12906 | BRI |
| *A. wallichii* | 16,1325 | 107,7378 | Averyanov & al. HAL10928 | MO |
| *A. wallichii* | 17,68583 | 105,765 | Averyanov & al. HAL11708 | MO |
| *A. wallichii* | 17,69722 | 105,765 | Averyanov & al. HAL12249 | MO |
| *A. wallichii* | 11,73111 | 109,1431 | Regalado & al. HLF4362 | MO |
| *A. wallichii* | 11,62722 | 105,8792 | Thai & al. HLF7743 | MO |
| *A. wallichii* | -15,8 | 145,3 | Forster PIF17298 | BRI |
| *A. wallichii* | 14,65 | 104,4167 | Averyanov VH5185 | MO database |
| *A. wallichii* | 12,5 | 108,5 | Averyanov VH6095 | MO database |
| *A. wallichii* | -16,0864 | 145,4617 | Jones s.n. | CANB |
| *A. wallichii* | -7,3333 | 146,45 | Craven & Schodde 1404 | CANBR |
| *A. wallichii* | -3,3667 | 142,1 | Clements 9625 | CANBR |
| *A. wallichii* | -6,4017 | 143,2169 | Clements & al. 10125 | CANBR |
| *A. wallichii* | -17,7728 | 145,65 | Ford 3287 | CANBR |
| *A. wallichii* | -16,4667 | 145,3667 | Jones 8714 | CANBR |
| *A. wallichii* | -18,2167 | 145,9 | Jones & al. 15715 | CANBR |
| *A. wallichii* | -16,7036 | 145,45 | Jones & Gray 18191 | CANBR |
| *A. wallichii* | -18,3667 | 146,25 | Mackenzie 046/98 | CANBR |
| *A. wallichii* | -14,2833 | 143,0667 | Roberts DLJ12906 | CANBR |
| *N. borneensis* | -1,66 | 108,84 | Ismail & al. 456 | K |
| *N. borneensis* | 4,6575 | 114,52 | Wong 939 | K |
| *N. borneensis* | 4,633333 | 114,5667 | Forman & Blewett 1113 | K |
| *N. borneensis* | 6,149167 | 116,5675 | Lugas 1267 | K |
| *N. borneensis* | 6 | 116,6667 | Beaman & al. 7404 | K |
| *N. borneensis* | 1,588889 | 110,1919 | Beaman & al. 11221 | K |
| *N. borneensis* | 4,654167 | 114,5494 | Kalat & al. 16915 | K |
| *N. borneensis* | 5,75 | 116,5 | Lamb 91574 | K |
| *N. veratrifolia* | -9,45778 | 159,93 | Dennis 12 | K |
| *N. veratrifolia* | -13,8436 | 167,4464 | Wheatley 344 | K |
| *N. veratrifolia* | 5,181944 | 100,5347 | Since coll. 469 | K |
| *N. veratrifolia* | 6,426667 | 116,7417 | Bakia 613 | K |
| *N. veratrifolia* | 4,303889 | 114,4444 | Simpson & Marsh 2103 | K |
| *N. veratrifolia* | -2,06667 | 147,05 | Sands & al. 2589 | K |
| *N. veratrifolia* | 13,26222 | 120,995 | Ramos & Edano 3271 | K |
| *N. veratrifolia* | 8,795 | 117,7033 | Co 3407 | K |
| *N. veratrifolia* | -6,19389 | 134,5497 | Buwalda 5065 | K |
| *N. veratrifolia* | -6,43194 | 143,3142 | Reeve 5208 | K |
| *N. veratrifolia* | 4,303889 | 114,4444 | Sands & al. 5495 | K |
| *N. veratrifolia* | 2,8175 | 99,63417 | Bartlett 6439 | K |
| *N. veratrifolia* | -1,00917 | 133,95 | Johns & al. 7710 | K |
| *N. veratrifolia* | -6,71667 | 145 | Takeuchi 11770 | K |
| *N. veratrifolia* | -9,68861 | 159,7181 | Fa'arodo & al. 12256 | K |
| *N. veratrifolia* | -7,32667 | 147,125 | Takeuchi & Towati 14644 | K |
| *N. veratrifolia* | -7,305 | 147,1333 | Takeuchi & al. 15096 | K |
| *N. veratrifolia* | -7,3056 | 147,1333 | Takeuchi 15096 | CANBR |
| *N. veratrifolia* | 3,816806 | 102,4164 | Henderson 24054 | K |
| *N. veratrifolia* | -7,41667 | 147,1667 | Streimann 24478 | K |
| *N. veratrifolia* | -9,08333 | 147,5667 | Isles & Vinas 34445 | K |
| *N. veratrifolia* | -2,15833 | 146,5639 | Kerenga & al. 77575 | K |
| *N. veratrifolia* | 1,588889 | 110,1919 | Beaman 11227b | K |
| *N. veratrifolia* | 2,8175 | 99,63417 | Barlett & Rue 76 | BM |
| *N. zollingeri* | 22,25 | 114,2 | Barretto 196 | K |
| *N. zollingeri* | 6,1 | 116,6667 | Andau 248 | K |
| *N. zollingeri* | 6,116667 | 116,7167 | Tadong 469 | K |
| *N. zollingeri* | 6 | 116,6667 | Beaman & al. 7416 | K |
| *N. zollingeri* | 6,345278 | 116,5572 | Beaman & al. 8782 | K |
| *N. zollingeri* | 2,042778 | 103,5611 | Holtman 10856 | K |
| *N. zollingeri* | 1,602222 | 103,5461 | Since coll. 12154 | K |
| *N. zollingeri* | 6,036944 | 116,6856 | Wood & Charrington 16370 | K |
| *N. zollingeri* | -7,53333 | 112,0667 | Raynal 18915 | K |
| *N. zollingeri* | 3,104722 | 105,6556 | Handusen 20407 | K |
| *N. zollingeri* | 5,48 | 100,26 | Saw & al. 44613 | K |
| *N. zollingeri* | 5,633333 | 117,2 | Rahim & al. 93285 | K |
| *N. zollingeri* | 4,956944 | 118,165 | Joseph & al. 120876 | K |
| *N. zollingeri* | 5,635278 | 117,1258 | Kulip & al. 133369 | K |
| *N. zollingeri* | 5,031944 | 118,3447 | Perumal & Dewol 135041 | K |
| *N. zollingeri* | 11,73111 | 109,1431 | J.C. Regalado & et al. HLF4415 | MO |
| *N. zollingeri* | 12,24528 | 109,1833 | Evrard s.n. | P |
| *N. zollingeri* | 17,4025 | 104,8089 | J.E. Vidal s.n. | P |
