# Supplementary material for: Evolution of the climatic tolerance and postglacial range changes of the most primitive orchids (Apostasioideae) within Sundaland, Wallacea and Sahul
Source: PeerJ. 2016 Aug 31;4:e2384. doi: 10.7717/peerj.2384 (PMC5012329; doi:10.7717/peerj.2384)
Supplement: Table S2 [file peerj-04-2384-s002.doc]

S2 Table. Genbank accession numbers of the sequences used in phylogenetic analysis.

| Species | matK | trnL | ITS |
| --- | --- | --- | --- |
| *Apostasia wallichii* | AY557212 | AY557219 | AY557228 |
| *Apostasia odorata* | AY557213 | AY557220 | AY557229 |
| *Apostasia nuda* | AY557214 | AY557221 | AY557230 |
| *Neuwiedia zollingeri* var. *javanica* | AY557210 | KC172542 | AY557226 |
| *Neuwiedia veratrifolia* | AY557211 | AY557218 | AY557227 |
| *Neuwiedia borneensis* | AY557209 | AY557216 | AY557225 |
