# Supplementary figures and images for: Evolution of the climatic tolerance and postglacial range changes of the most primitive orchids (Apostasioideae) within Sundaland, Wallacea and Sahul
Source: PeerJ. 2016 Aug 31;4:e2384. doi: 10.7717/peerj.2384 (PMC5012329; doi:10.7717/peerj.2384)

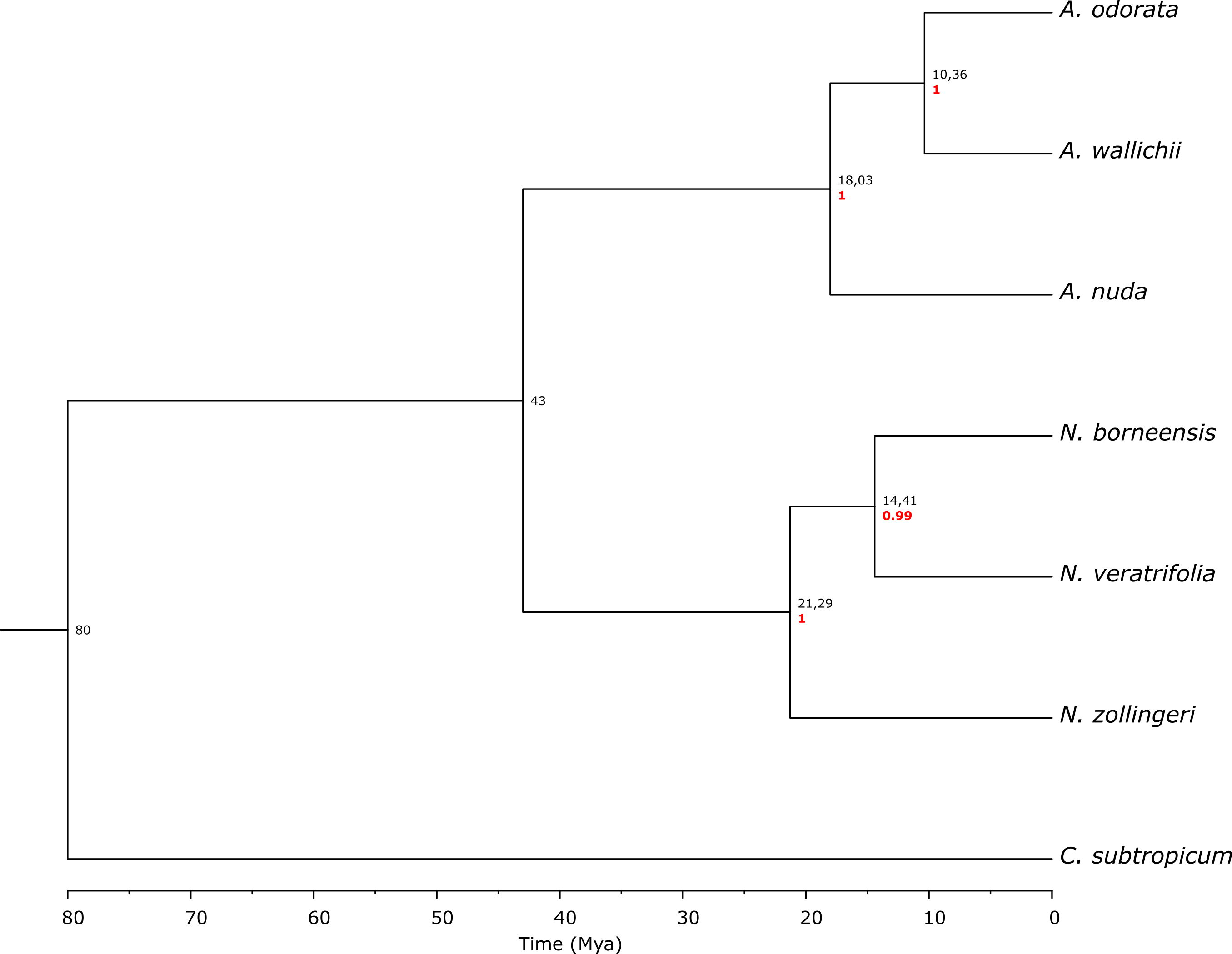

Supplement: Figure S1 — Bold text in red indicates posterior probability values and black text indicates estimated divergence times. [file peerj-04-2384-s003.png]
